# The Brazilian Initiative on Precision Medicine (BIPMed): fostering genomic data-sharing of underrepresented populations

**DOI:** 10.1038/s41525-020-00149-6

**Published:** 2020-10-02

**Authors:** Cristiane S. Rocha, Rodrigo Secolin, Maíra R. Rodrigues, Benilton S. Carvalho, Iscia Lopes-Cendes

**Affiliations:** 1 Department of Medical Genetics and Genomic Medicine, School of Medical Sciences, University of Campinas (UNICAMP), Campinas, SP Brazil; 2 The Brazilian Institute of Neuroscience and Neurotechnology (BRAINN), University of Campinas (UNICAMP), Campinas, SP Brazil; 3 Department of Statistics, Institute of Mathematics, Statistics, and Scientific Computing, University of Campinas (UNICAMP), Campinas, SP Brazil

**Keywords:** Molecular medicine, Medical genomics

## Abstract

The development of precision medicine strategies requires prior knowledge of the genetic background of the target population. However, despite the availability of data from admixed Americans within large reference population databases, we cannot use these data as a surrogate for that of the Brazilian population. This lack of transferability is mainly due to differences between ancestry proportions of Brazilian and other admixed American populations. To address the issue, a coalition of research centres created the Brazilian Initiative on Precision Medicine (BIPMed). In this study, we aim to characterise two datasets obtained from 358 individuals from the BIPMed using two different platforms: whole-exome sequencing (WES) and a single nucleotide polymorphism (SNP) array. We estimated allele frequencies and variant pathogenicity values from the two datasets and compared our results using the BIPMed dataset with other public databases. Here, we show that the BIPMed WES dataset contains variants not included in dbSNP, including 6480 variants that have alternative allele frequencies (AAFs) >1%. Furthermore, after merging BIPMed WES and SNP array data, we identified 809,589 variants (47.5%) not present within the 1000 Genomes dataset. Our results demonstrate that, through the incorporation of Brazilian individuals into public genomic databases, BIPMed not only was able to provide valuable knowledge needed for the implementation of precision medicine but may also enhance our understanding of human genome variability and the relationship between genetic variation and disease predisposition.

## Introduction

Precision medicine combines molecular and clinical information to improve healthcare delivery. Since precision medicine uses individualised information from patients, such as genomic signatures, it allows for more accurate diagnoses and tailored treatment options^1,2^. This approach is a significant improvement over the current paradigm in which physicians prescribe therapeutics designed to most effectively treat the average patient. However, precision medicine cannot be implemented without understanding the contribution of human genomic diversity to health and disease^3^. Therefore, the development of strategies used in precision medicine requires detailed knowledge of the genetic background of the population throughout which it will be applied. This approach is particularly important because the distribution of rare and common variants may differ depending on the population considered^4–8^. This issue is more challenging for admixed American populations since their genomes present a mosaic of chromosomal tracts derived from different ancestral populations^9–11^.

Large-scale genomic studies conducted using subjects not selected based on disease-related phenotypes (defined here as the reference population) have been performed to characterise the genetic architecture of specific populations. These studies include the HapMap project^12^, 1000 Genomes Project^4^, Simons Genome Diversity Project^13^, and Genome Aggregation Database (gnomAD)^14^. More recently, national initiatives devoted to the development and improvement of precision medicine have been conducted in several countries, including the United States^15^, the United Kingdom^16^, the Netherlands^17^, Qatar^18^, Japan^19^, Australia^20^, and some African countries^21^. Several of the projects relied on the findings of previous large-scale genomic studies to guide experimental design and analytical protocols, highlighting the importance of acquiring genomic information at the population level to facilitate the implementation of precision medicine.

However, despite the availability of reference genomes from some admixed American populations, this population group remains underrepresented in all large reference population databases, and especially in publicly available datasets^22^. For instance, we found that of the 2504 individuals who participated in the 1000 Genome Project and 141,456 individuals included in the gnomAD v2.1 dataset, only 20.13% and 12.53% were admixed Americans, respectively. Even though Brazil has the largest population among all countries in Latin America and the Caribbean (32.57% in 2015) and is the fifth-largest population worldwide (https://population.un.org/wpp/Download/Standard/Population/), the Brazilian population is underrepresented in both public genomic reference databases and genome-wide association studies (GWAS). This observation remains true even if one includes Latin American populations represented in worldwide collaborative studies, such as the 1000 Genomes Project and gnomAD, which involved Colombian, Peruvian, Puerto Rican, and Mexican populations^4,14,22^. Indeed, among the 3529 studies published in the GWAS catalogue^3^, only 75 studies contain data from Brazilian individuals, and only three are exclusively comprised of Brazilian populations^23–25^.

Similar to other admixed American populations, the Brazilian population is derived from sub-Saharan African, European, and Native American populations^25–28^. However, we cannot use other admixed American populations as a reference for Brazilians due to differences in the proportions of ancestral populations from which the current Brazilian and other admixed Americans are derived^9–11,28,29^. In this specific case, genomic markers detected in other admixed American populations have the potential to mischaracterise the genomic landscape of interest because the allele frequencies of some genetic markers are population-specific. In addition, due to the size and heterogeneous background of the Brazilian population, different ancestral proportions are likely to occur in different geographic regions of the country as a result of evolutionary and demographic events^26,27^. Although previous reports have included genomic information from Brazilian populations, the limited quantity of variant information across the genome^27^ and the restricted set of subpopulations evaluated are insufficient^25,26^, and a greater volume of genomic data will be needed for the adequate implementation of precision medicine in Brazil.

Importantly, data generated in the majority of previous studies that have examined the Brazilian population are not publicly available. To address the issue mentioned above, a coalition of five research, dissemination, and innovation centres supported by the São Paulo Research Foundation (http://www.fapesp.br/) created the Brazilian Initiative on Precision Medicine (BIPMed; http://www.bipmed.org) in November of 2015. The main objective of the BIPMed project is to facilitate the implementation of precision medicine in Brazil by acting as a catalytic element used to foster collaboration among stakeholders, which include physicians, scientists, health authorities, policymakers, and society. In this context, we aim to investigate the distribution of rare and common variants present in two BIPMed datasets and assess the composition of a sample of the Brazilian population from a large metropolitan area in São Paulo, the most populated state of Brazil, located in the southeast region of the country. In the current manuscript, we present evidence highlighting the importance of compiling and analysing genomic datasets from underrepresented populations in the context of genomic and precision medicine. We initially describe the two datasets available in BIPMed: a whole-exome sequence (WES) dataset and a single nucleotide polymorphism (SNP) array genotyping dataset. Second, we present a comparison of variants identified from each dataset against those of publicly available databases. Finally, we compared the population genomic structure provide by information derived from WES and SNP array data.

## Results

### WES dataset

Overall, we found 851,109 different variants within 18,202 genes in the dataset, which included single nucleotide variants and small insertions and deletions. After removing variants containing >20% missing data, 823,481 variants remained. Among these, 522,290 (63.4%) had alternative allele frequency values (AAF) < 1%, and 96,971 (11.8%) were not present in the dbSNP database. Among the variants absent from the dbSNP, 6480 had AAF values >1% (Supplementary Data). Interestingly, nine variants absent from the dbSNP occurred at a high frequency within the BIPMed dataset (>90%).

A comparison between the WES dataset and the Clinvar database revealed that 727 variants were classified as pathogenic and 41 were likely to be pathogenic. Among these, we identified 509 (70.0%) pathogenic variants and 33 (80.5%) variants that were likely to be pathogenic that were rare (AAF < 1%) in the BIPMed WES dataset. The AAF values of most of the common variants (AAF ≥ 1%) found in the WES dataset were similar to those identified using gnomAD and TOPMed from the dbSNP dataset. Interestingly, we did not find variants classified as pathogenic in the BIPMed WES data that overlapped with the 1000 Genome dataset.

### SNP array dataset

After performing quality control procedures, the SNP array data contained 902,939 variants; 25,492 of which overlapped with WES data, and 897,990 (99.44%) were also determined to be present in the 1000 Genomes datasets. We identified 65,519 variants with AAF values between 1 and 5%, and 831,266 with AAF values >5%.

### Comparing genomic population structure between WES and SNP array datasets

**Fig. 1:**
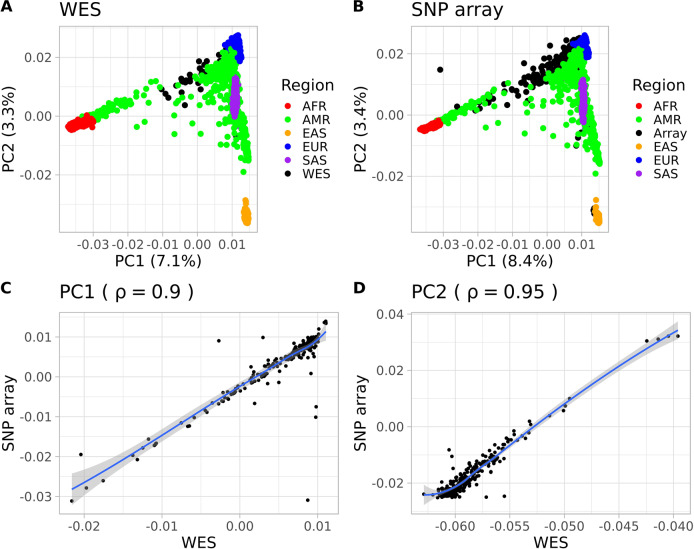
Comparison between principal components (PCs) of WES and SNP array data. **a**, **b** Scatterplots indicate the two first principal components identified using WES and SNP array datasets (black) and 1000 Genome populations, including Europeans (EUR = blue), sub-Saharan Africans (AFR = red), admixed Americans (AMR = green), East Asians (EAS = orange), and South Asians (SAS = purple). **c**, **d** Correlation plots assessing the relationship between the WES and SNP array for PC1 (**a**) and PC2 (**b**). Each point represents one individual. Solid lines indicate the best fit of the data via local regression (LOESS) with a 95% confidence interval shown by the grey area.

**Fig. 2:**
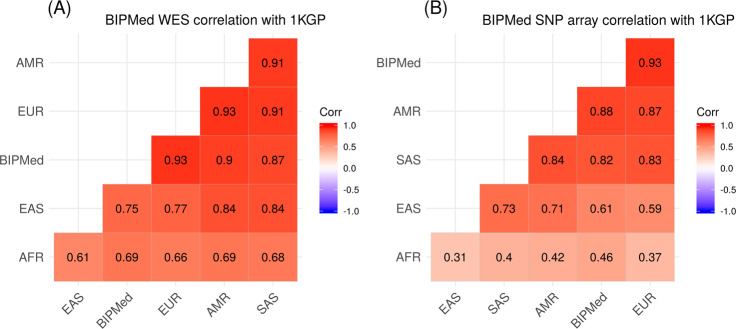
Comparison of Euclidean distance estimations between the BIPMed datasets and continental populations from the 1000 Genome project (1 KGP). Estimates were based on minor allele frequency (MAF) from BIPMed WES (**a**) and SNP array data (**b**). The red and blue colours in the legend indicate positive and negative correlations between two populations, respectively. Values closer to 1.0 indicate a more significant correlation between the two populations. AFR Sub-Saharan Africans, AMR Admixed Americans, EAS East Asians, EUR Europeans, SAS South Asians.

The PCA used to assess the two BIPMed datasets revealed that both WES and SNP array datasets produced similar results, which are in accordance with previous reports^25–28^. PC1 shows variant frequencies similar to European populations, and PC2 indicates characteristics of both European and sub-Saharan African populations (Fig. 1a, b). In addition, the similarity between both PCA performed in the two BIPMed datasets reflected in high correlation estimations of WES and SNP array data for PC1 (ρ ≥ 0.90; Fig. 1c) and PC2 (ρ ≥ 0.95; Fig. 1d). According to Euclidean distance estimations, both WES (Fig. 2a) and the SNP array (Fig. 2b) were closest to the European population, followed by admixed American populations.

### Comparing the BIPMed dataset with the 1000 Genomes dataset

To compare the allele frequency of variants found within the BIPMed dataset with the 1000 Genomes dataset, we first merged the WES and SNP array to produce a single, large dataset, which provided 1,626,829 unique autosomal variants from both the SNP array and WES. Allele frequencies were estimated, based on merged WES and SNP array data. The estimation revealed 1,136,454 (69.9%) common variants with a minimum allele frequency (MAF) ≥ 1% and 490,375 (30.1%) rare variants with a MAF < 1%. After applying genotype and individual filtering^30^, 817,240 (52.5%) autosomal variants could be found in the 1000 Genomes database. These results indicated that 809,589 variants (47.5%) present in the BIPMed reference population were not present in the 1000 Genomes datasets.

After performing a comparison of BIPMed data with the 1000 Genomes datasets, we found that rare variants in European (75,584; 9.2%), sub-Saharan African (67,109; 8.2%), and admixed American populations (34,360; 4.2%) were common in the BIPMed database. In contrast, 7493 (1.0%) common variants in European populations, 65,565 (8.0%) in sub-Saharan African, and 12,132 (1.5%) in admixed American populations were determined to be rare in the BIPMed reference datasets (Table 1). Assuming the null hypothesis that there is a similarity between the frequency of variants in the BIPMed and the 1000 Genomes datasets, our results provide evidence that data are not compatible with the null hypothesis (Fisher’s exact test *p* value = 2.2e^−16^). It is important to point out that the BIPMed sample (*N* = 358), was similar in size to the other datasets used for the comparative analyses performed in the present work, which contained European (*N* = 404), African (*N* = 504), and Admixed American (*N* = 347) populations.

**Table 1:** Distribution of minimum allele frequencies (MAF) among variants.

MAF distribution	Common in BIPMed	Rare in BIPMed	Total overlap	*P* value
Common in EUR	595,874 (72.9%)	7493 (1.0%)	817,240 (100%)	2.2e^−16^
Rare in EUR	75,584 (9.2%)	138,289 (16.9%)		
Common in AFR	604,349 (74.0%)	65,565 (8.0%)	817,240 (100%)	2.2e^−16^
Rare in AFR	67,109 (8.2%)	80,217 (9.8%)		
Common in AMR	637,098 (78.0%)	12,132 (1.5%)	817,240 (100%)	2.2e^−16^
Rare in AMR	34,360 (4.2%)	133,650 (16.3%)		

The 817,240 variants are classified by their population of origin and include Europeans (EUR), Africans (AFR), and admixed Americans (AMR), according to the 1000 Genomes Project. We defined common variants as those with a MAF higher than or equal to 0.01, and rare variants otherwise. We counted total variants produced using high-density SNP genotyping and whole-genome sequencing and removed high-density SNP genotypes with MAF values less than 0.01 to avoid bias from genotyping errors. *P* values were calculated using Fisher’s exact test.

## Discussion

The application of precision medicine in admixed American populations requires a refined knowledge of the environmental exposure, lifestyle, biological susceptibility, and genomic structure of their admixed genomes^15,31^. Indeed, studies have shown that risk-associated allele frequencies of different populations vary, a phenomenon which implies that risk-associated alleles identified in one population are not necessarily informative when predicting disease prevalence of all human groups^7,8^. If physicians do not take this information into account when implementing precision medicine, they are likely to provide incorrect diagnoses of patients, and correspondingly, provide inadequate treatments^32^. This scenario is especially likely to occur in Brazilian admixed populations, which are remarkably underrepresented in public genomic databases^28^.

Here we aimed to highlight the importance of compiling, analysing, and sharing genomic data obtained from an underrepresented population to enhance the application of precision medicine. Also, we have shown that, even when limited datasets are available, they can be of value in this scenario, since small datasets are better than no information at all. This point is particularly relevant for scientists and physicians in mid-low-income countries, which often believe that new developments in precision medicine may not be of use to the populations they serve.

The sample studied was representative of the target population; patients followed at the University of Campinas (UNICAMP) hospital, which was the population from the geographic region delimited by our study. However, based on the limited public data available in the Brazilian population^25–28^, it is very likely that multiple datasets from different geographic regions will be needed to generate data for the application of precision medicine in the different areas of Brazil. This observation is a very relevant point, which is probably valid for many other regions, if not all the Americas, given the remarkable differences observed between population histories. These differences are based on the various origins of founder populations, migration waves, and other population genetics phenomena. Therefore, we strongly believe in the value of presenting BIPMed data, which contributes to this type of discussion, which is relevant to any country with diverse and admixed populations.

In the BIPMed WES dataset, we identified 768 variants classified as pathogenic or likely pathogenic, according to Clinvar. This result could have a significant impact on disease risk estimates for the Brazilian population. In addition, we observed that 47.5%% of the variants present in the BIPMed dataset were not present in the 1000 Genomes database, including 6480 variants with AAF values that were higher than 1% in BIPMed. Indeed, these novel variants have the potential to reveal new insights regarding genetic variation and the effects of complex traits in admixed Brazilians. However, we are aware that validation by other techniques, such as Sanger sequencing, will be needed to confirm the presence of the identified variants and exclude the possibility that they are false positives generated by the WES technique. Validation is especially important for the nine variants that are absent from dbSNP but appeared at a high frequency in BIPMed (>90%).

Other potential causes for the divergence observed in allele frequencies reported here should also be considered, such as the technical differences between WES (BIPMed) and whole-genome sequencing (WGS) platforms (1000 Genomes project). In this case, bias and variability may be affected by the use of different sequencing equipment and libraries for exome capture, which covers different genomic regions.

Given the fact that the BIPMed reference databases provide two different types of genomic information for 239 individuals, we could also compare whether the two datasets produced similar estimates of population structure. Our results showed that, based on the first two principal components (which possessed the highest proportion of variability observed), WES and SNP array datasets provided similar information regarding the genomic structure (Fig. 1). The concordance between the two datasets was important since results obtained with the SNP array could have had a European bias^33^. However, since the data generated by WES covered all coding regions, and therefore was not at risk of bias, the concordance of results produced independently using the different platforms validates our results. Previous studies also compared WES and SNP array datasets from individuals that were predominantly from the Middle East, North Africa, Western Europe, and five admixed American individuals from Brazil, Colombia, and Mexico. They demonstrated that WES could provide population structure adjustments that were similar to those produced using SNP array data^34^. Interestingly, Euclidean distances determined only reflected the structure observed in PC1, in which BIPMed data was closer to that of European and admixed American/Asian, rather than African populations. In this case, we suggest that the Euclidean distance estimates are less robust than eigenvector and eigenvalue estimations from the PCA, and thus, provide limited information regarding genomic structure.

The value of describing the BIPMed datasets can be further highlighted, since they provide a complete genomic map of variants within admixed Brazilian individuals, as BIPMed contains data that is rich in variant information found within the coding regions from WES, and additional information from the noncoding genomic regions provided by SNP array genotyping. By assessing the similarity between the frequencies of all variants identified by WES and SNP arrays in BIPMed and 1000 Genomes datasets, we found that they differ significantly. This result indicates that none of the admixed American populations present in the 1000 Genomes dataset can be used as a surrogate for studies of the Brazilian population since the 1000 Genomes datasets produced significantly different allele frequencies for both common and rare variants than the BIPMed datasets. Nevertheless, we acknowledge that the 1000 Genome dataset was built from WGS, which includes all variants within the genome. Indeed, differences in NGS platforms may influence our results because we did not evaluate all variants available in the 1000 Genome database.

Similar to BIPMed, other Brazilian initiatives have aimed to make genomic data more transparent and reproducible^35^. However, BIPMed is the first to provide the public with easy access to raw data (https://bipmed.org/datasharing/). Additionally, the data-sharing process in BIPMed has been facilitated by the federated model of genomic databases proposed and provided by the Global Alliance for Genomics and Health^36^.

However, we are aware of the limitations of the data currently available in BIPMed. First, although we analysed individuals born in all five geographic regions of Brazil (Table 2), BIPMed samples were predominantly from the Southeast region (49.44%), followed by the Northeast (5.59%), and the South (2.79%). Therefore, we can only provide reliable genomic estimates of population structure for three of the five Brazilian geographic regions. Second, the BIPMed dataset does not contain all genome variations, and likely missed rare variants located outside coding regions and structural variants. However, the latter can be assessed based on the SNP array data provided. Both limitations are currently being addressed by expanding the geographic reach of BIPMed samples and by including whole-genome data from the Brazilian reference individuals. We also encourage other Brazilian research groups to help improve the BIPMed database by depositing data generated from individuals from different geographic regions of Brazil (https://bipmed.org/docs/2_DepositDataBIPMed.docx).

**Table 2:** Distribution of birth location of BIPMed reference individuals within five Brazilian geographic regions.

Brazilian region	Number of individuals
North	1 (0.24%)
Northeast	20 (5.59%)
Centre West	3 (0.84%)
Southeast	177 (49.44%)
South	10 (2.29%)
Unknown	147 (41.06%)

To date, BIPMed includes eight public databases, which contain information from 884 Brazilian admixed individuals distributed among six disease-specific datasets, and the two reference datasets included in this report. Though the disease-specific datasets in BIPMed do not include WES or SNP array data, BIPMed has provided valuable information for the application of precision medicine within the Brazilian admixed population.

One additional challenge in the implementation of precision medicine is related to the integration and sharing of genomic and clinical data generated by different groups and interested parties^36,37^. The worldwide community, including the research community, would benefit significantly from increased cooperation. It will enhance the expansion and improve the availability of datasets, facilitating the detection of smaller genetic effects in complex disorders. It is well understood that the ability to access increased quantities of shared genomic and clinical data improves our understanding of the mechanisms underlying the diseases that affect individuals worldwide, and these diseases may have population-specific features. Through networking, clinicians will have access to improved information for performing risk assessment, prevention, and the delivery of optimised treatment regimens. Thus, in addition to its local importance for the full implementation of precision medicine in Brazil, we expect that BIPMed will catalyse similar initiatives within other underrepresented populations worldwide.

In conclusion, we showed that by studying two BIPMed datasets that included information from reference admixed Brazilian individuals from a specific geographic area, we detected a diverse population background, even when compared with other admixed American populations. The population structure estimations provided by WES and SNP array data were concordant. By incorporating admixed Brazilian individuals in public genomic databases, BIPMed not only contributes important knowledge for the proper implementation of precision medicine in Brazil, but it also enhances information regarding the variability of the human genome and the relationship between genetic variation and predisposition to diseases.

## Methods

### Subjects

We examined 358 individuals, predominantly from Southeast Brazil (49.44%; Table 2), at the University of Campinas (UNICAMP, Campinas, Brazil). BIPMed participants were identified among people who were accompanying patients in the out-patient clinic of our hospital and were mainly unrelated spouses of patients. We also applied a structured questionnaire regarding serious health issues and excluded individuals that were known to have major health problems.

Genomic DNA was obtained from peripheral blood via the phenol–chloroform procedure^38^. DNA samples were evaluated using a Qubit^®^ 2.0 Fluorometer (Invitrogen, Carlsbad, CA, USA) and an Epoch 2 microplate spectrophotometer (BioTek Instruments Inc., Winooski, VT, USA). The present study was approved by the Research Ethics Committee at UNICAMP, and all participants signed consent forms before participating in the study.

### WES dataset

DNA samples from 257 of the 358 individuals were fragmented using Covaris^®^ sonicator equipment (Covaris Company, Woburn, MA, USA). Fragmented DNA was end-repaired, and adapters were added using the SurSelect Human All Exon V5 target enrichment technique (Agilent Technologies, Santa Clara, CA, USA). Exome libraries were prepared following the standard Illumina protocol for paired-end sequencing (Illumina Inc., San Diego, CA, USA). Library quality was evaluated using Bioanalyzer DNA High Sensitivity chips (Agilent Technologies, Santa Clara, CA). Sequencing was performed on the Illumina HiSeq2500 platform with 100 base-pair reads. We aligned paired reads using BWA-MEM v0.7.12^39^. Picard Tools v2.5.0 (http://broadinstitute.github.io/picard) was used for marking duplicates and indexing. Local realignment, quality base re-calibration, and variant calling were performed with the Genome Analysis Toolkit v4.0^40^.

### SNP array dataset

Genotype calling from 340 of 358 individuals was performed using the Genome-Wide Human SNP Array 6.0 platform (Affymetrix Inc, Santa Clara, CA) in the Multiuser Equipment Facility at UNICAMP. The genotype was called from fluorescent signals observed using the CRLMM package^41^ in R software (https://www.r-project.org/) and converted to the variant calling format file by in-house Perl scripts.

### Data analysis

We removed genotypes in which more than 20% of genomic data were missing (missing data >20%) from the WES dataset. Since the genotype call rate from CRLMM was 100%, we did not need to filter the SNP array as a result of missing data. We calculated the AAF and minor allele frequency (MAF) of variants from both WES and SNP array data. Variants from the SNP array with a MAF < 0.01 were removed to avoid bias due to genotyping errors from the array technique^30^. We defined rare variants as those with allele frequencies <1% and common variants were defined as those that occurred at frequencies ≥1%^4^. To investigate the presence of pathogenic variants in WES, we compared WES data with Clinvar version 20190211^42^. Additionally, we compared the distribution of rare and common pathogenic/likely pathogenic variants within WES data with distributions determined using the 1000 Genome Project, gnomAD, and TOPMed databases^4,14,43,44^. These data analyses were performed using VariantAnnotation^45^, vcfR^46^, and ggplot2^47^ packages from Bioconductor, and in-house scripts in R software.

### Genomic structure estimates using different datasets

To evaluate the estimates of the genomic structure of the BIPMed samples obtained with WES and SNP array data, we compared the two first principal components (PCs) produced from assessing the 239 individuals with available WES and SNP array data. First, we filtered each dataset via Hardy–Weinberg disequilibrium (*p* value < 0.01) and merged each separately with the 1000 Genome dataset. After dataset merging, we pruned variants that had linkage disequilibrium values (window size = 50 SNPs, shift step = 5 SNPs, and *r*^2^ = 0.5) and estimated PCs via PCA. We also calculated Euclidean distances based on MAF between the populations of the datasets and 1000 Genome Project to investigate genomic structure using a different estimation method. All filtering, dataset merging, and PCA were performed using PLINK v1.9 software^48^. We estimated the Pearson’s correlation between WES and SNP array data based on the two first principal components using the R software.

### Reporting summary

Further information on research design is available in the Nature Research Reporting Summary linked to this article.

## Supplementary information

Reporting Summary

Supplementary Data

## Data Availability

The datasets generated during and/or analysed during the current study are available in the GEO repository (SNP-array dataset: https://www.ncbi.nlm.nih.gov/geo/query/acc.cgi?acc=GSE156652) and at the EVA repository (WES-dataset) under the following accessions: Project: PRJEB39251 Analyses: ERZ1463065, as well as in the BIPMed repository, http://bipmed.iqm.unicamp.br/genes and http://bipmed.iqm.unicamp.br/snparray/genes.

## Code availability

All Custom codes used in the generation or processing of datasets are available at https://github.com/crirocha/BIPMed/ and https://github.com/labbcb/bipmed-analysis/blob/master/BIPMed_analysis.md.

## References

[1] Aronson SJ, Rehm HL (2015). Building the foundation for genomics in precision medicine. Nature.

[2] Hindorff LA (2018). Prioritizing diversity in human genomics research. Nat. Rev. Genet.

[3] MacArthur J (2017). The new NHGRI-EBI Catalog of published genome-wide association studies (GWAS Catalog). Nucleic Acids Res.

[4] Auton A (2015). A global reference for human genetic variation. Nature.

[5] Altshuler DM (2010). Integrating common and rare genetic variation in diverse human populations. Nature.

[6] Casals F, Bertranpetit J (2012). Genetics. Human genetic variation, shared and private. Science.

[7] Moonesinghe R (2012). Estimating the contribution of genetic variants to difference in incidence of disease between population groups. Eur. J. Hum. Genet..

[8] Myles S, Davison D, Barrett J, Stoneking M, Timpson N (2008). Worldwide population differentiation at disease-associated SNPs. BMC Med Genomics.

[9] Adhikari K, Mendoza-Revilla J, Chacón-Duque JC, Fuentes-Guajardo M, Ruiz-Linares A (2016). Admixture in Latin America. Curr. Opin. Genet. Dev..

[10] Homburger, J. R. et al. Genomic Insights into the Ancestry and Demographic History of South America. *PLoS Genetics* **11**10.1371/journal.pgen.1005602 (2015). 10.1371/journal.pgen.1005602 PMC4670080 26636962

[11] Ruiz-Linares A (2014). Admixture in Latin America: geographic structure, phenotypic diversity and self-perception of ancestry based on 7,342 individuals. PLoS Genet..

[12] The International HapMap Consortium. (2003). The International HapMap Project. Nature.

[13] Mallick S (2016). The Simons Genome Diversity Project: 300 genomes from 142 diverse populations. Nature.

[14] Lek M (2016). Analysis of protein-coding genetic variation in 60,706 humans. Nature.

[15] Collins FS, Varmus H (2015). A new initiative on precision medicine. N. Engl. J. Med.

[16] Walter K (2015). The UK10K project identifies rare variants in health and disease. Nature.

[17] Genome of the Netherlands Consortium. (2014). Whole-genome sequence variation, population structure and demographic history of the Dutch population. Nat. Genet.

[18] Fakhro KA (2016). The Qatar genome: a population-specific tool for precision medicine in the Middle East. Hum. Genome Var..

[19] Yamaguchi-Kabata Y (2015). iJGVD: an integrative Japanese genome variation database based on whole-genome sequencing. Hum. Genome Var..

[20] Williamson, R. et al. *The future of precision medicine in Australia*. (Australian Council of Learned Academies (ACOLA), 2018).

[21] Rotimi C (2014). Enabling the genomic revolution in Africa. Science.

[22] Popejoy AB, Fullerton SM (2016). Genomics is failing on diversity. Nature.

[23] Deng X (2013). Genome wide association study (GWAS) of Chagas cardiomyopathy in *Trypanosoma cruzi* seropositive subjects. PLoS One.

[24] Ledda M (2014). GWAS of human bitter taste perception identifies new loci and reveals additional complexity of bitter taste genetics. Hum. Mol. Genet.

[25] Mychaleckyj JC (2017). Genome-wide analysis in Brazilians reveals highly differentiated native American genome regions. Mol. Biol. Evolution.

[26] Kehdy FSG (2015). origin and dynamics of admixture in Brazilians and its effect on the pattern of deleterious mutations. Proc. Natl Acad. Sci..

[27] Rodrigues de Moura R, Coelho AVC, de Queiroz Balbino V, Crovella S, Brandão LAC (2015). Meta-analysis of Brazilian genetic admixture and comparison with other Latin America countries. Am. J. Hum. Biol..

[28] Secolin R (2019). Distribution of local ancestry and evidence of adaptation in admixed populations. Sci. Rep..

[29] Chacón-Duque JC (2018). Latin Americans show wide-spread Converso ancestry and imprint of local native ancestry on physical appearance. Nat. Commun..

[30] Anderson CA (2010). Data quality control in genetic case-control association studies. Nat. Protoc..

[31] Schumann G (2019). Precision medicine and global mental health. Lancet Glob. Health.

[32] Martin AR (2017). Human demographic history impacts genetic risk prediction across diverse populations. Am. J. Hum. Genet..

[33] Nielsen R (2004). Population genetic analysis of ascertained SNP data. Hum. Genomics.

[34] Belkadi, A. et al. Whole-exome sequencing to analyze population structure, parental inbreeding, and familial linkage. *Proc. Nal Acad. Sc.* 201606460–201606460: 10.1073/pnas.1606460113 (2016). 10.1073/pnas.1606460113 PMC4914194 27247391

[35] Magalhães WCS (2018). EPIGEN-Brazil Initiative resources: a Latin American imputation panel and the Scientific Workflow. Genome Res.

[36] Global Alliance for Genomics and Health. (2016). GENOMICS. A federated ecosystem for sharing genomic, clinical data. Science.

[37] Cook-Deegan R, Ankeny RA, Maxson Jones K (2017). Sharing data to build a medical information commons: from Bermuda to the Global Alliance. Annu Rev. Genomics Hum. Genet.

[38] Sambrook, J., Fritsch, E. F. & Maniatis, T. *Molecular Cloning: a laboratory manual*. 2nd edn, 1659 (Cold Spring Harbor Laboratory, Cold Spring Harbor Laboratory Press, 1989).

[39] Li H, Durbin R (2009). Fast and accurate short read alignment with Burrows-Wheeler transform. Bioinformatics.

[40] McKenna A (2010). The Genome Analysis Toolkit: a MapReduce framework for analyzing next-generation DNA sequencing data. Genome Res.

[41] Scharpf RB, Irizarry RA, Ritchie ME, Carvalho B, Ruczinski I (2011). Using the R Package crlmm for Genotyping and Copy Number Estimation. J. Stat. Softw..

[42] Landrum MJ (2014). ClinVar: public archive of relationships among sequence variation and human phenotype. Nucleic Acids Res.

[43] Natarajan P (2018). Deep-coverage whole genome sequences and blood lipids among 16,324 individuals. Nat. Commun..

[44] Zekavat SM (2018). Deep coverage whole genome sequences and plasma lipoprotein(a) in individuals of European and African ancestries. Nat. Commun..

[45] Obenchain V (2014). VariantAnnotation: a Bioconductor package for exploration and annotation of genetic variants. Bioinformatics.

[46] Knaus BJ, Grünwald NJ (2017). vcfr: a package to manipulate and visualize variant call format data in R. Mol. Ecol. Resour..

[47] Wickham, H. *ggplot2: Elegant Graphics for Data Analysis*. 2 edn, (Springer International Publishing, 2016).

[48] Purcell S (2007). PLINK: a tool set for whole-genome association and population-based linkage analyses. Am. J. Hum. Genet..

