# Supplementary material for: The Brazilian Initiative on Precision Medicine (BIPMed): fostering genomic data-sharing of underrepresented populations
Source: NPJ Genom Med. 2020 Oct 2;5:42. doi: 10.1038/s41525-020-00149-6 (PMC7532430; doi:10.1038/s41525-020-00149-6)
Supplement: Supplementary file 1 — Reporting Summary [file 41525_2020_149_MOESM1_ESM.pdf]

nature research

Corresponding author(s): Iscia Lopes-Cendes, M.D., Ph.D.

Last updated by author(s): Mar 15, 2020

Reporting Summary

Nature Research wishes to improve the reproducibility of the work that we publish. This form provides structure for consistency and transparency in reporting. For further information on Nature Research policies, see Authors & Referees and the Editorial Policy Checklist.

Statistics

For all statistical analyses, confirm that the following items are present in the figure legend, table legend, main text, or Methods section.

n/a	Confirmed	
☐	☒	The exact sample size (*n*) for each experimental group/condition, given as a discrete number and unit of measurement
☐	☒	A statement on whether measurements were taken from distinct samples or whether the same sample was measured repeatedly
☐	☒	The statistical test(s) used AND whether they are one- or two-sided<br>*Only common tests should be described solely by name; describe more complex techniques in the Methods section.*
☐	☒	A description of all covariates tested
☐	☒	A description of any assumptions or corrections, such as tests of normality and adjustment for multiple comparisons
☐	☒	A full description of the statistical parameters including central tendency (e.g. means) or other basic estimates (e.g. regression coefficient) AND variation (e.g. standard deviation) or associated estimates of uncertainty (e.g. confidence intervals)
☐	☒	For null hypothesis testing, the test statistic (e.g. *F*, *t*, *r*) with confidence intervals, effect sizes, degrees of freedom and *P* value noted<br>*Give P values as exact values whenever suitable.*
☒	☐	For Bayesian analysis, information on the choice of priors and Markov chain Monte Carlo settings
☐	☒	For hierarchical and complex designs, identification of the appropriate level for tests and full reporting of outcomes
☐	☒	Estimates of effect sizes (e.g. Cohen's *d*, Pearson's *r*), indicating how they were calculated

Our web collection on statistics for biologists contains articles on many of the points above.

Software and code

Policy information about availability of computer code

Data collection	WES data was generated by sequencing, which was performed on the Illumina HiSeq2500 platform with 100 base-pair reads. We aligned paired reads using BWA-MEM v0.7.12. Picard Tools v2.5.0 (http://broadinstitute.github.io/picard) was used for marking duplicates and indexing. Local realignment, quality base re-calibration, and variant calling were performed with the Genome Analysis Toolkit v4.0. SNP data was obtained using the Genome-Wide Human SNP Array 6.0 platform (Affymetrix Inc, Santa Clara, CA) . The genotype was called from fluorescent signals observed using the CRLMM package in R software (https://www.r-project.org/) and converted to the variant calling format file by in-house Perl scripts.
Data analysis	Data analyses were performed using VariantAnnotation, vcfR , and ggplot2 packages from Bioconductor, and in-house scripts in R software. All filtering, dataset merging, and PCA were performed using PLINK v1.9 software. We estimated the Pearson's correlation between WES and SNP array data based on the two first principal components using the R software.

For manuscripts utilizing custom algorithms or software that are central to the research but not yet described in published literature, software must be made available to editors/reviewers. We strongly encourage code deposition in a community repository (e.g. GitHub). See the Nature Research guidelines for submitting code & software for further information.

Data

Policy information about availability of data

All manuscripts must include a data availability statement. This statement should provide the following information, where applicable:

- Accession codes, unique identifiers, or web links for publicly available datasets
- A list of figures that have associated raw data
- A description of any restrictions on data availability

The datasets generated during and/or analysed during the current study are available in the BIPMed repository, http://bipmed.iqm.unicamp.br/genes and http://bipmed.iqm.unicamp.br/snparray/genes

Field-specific reporting

Please select the one below that is the best fit for your research. If you are not sure, read the appropriate sections before making your selection.

☒ Life sciences ☐ Behavioural & social sciences ☐ Ecological, evolutionary & environmental sciences

For a reference copy of the document with all sections, see nature.com/documents/nr-reporting-summary-flat.pdf

Life sciences study design

All studies must disclose on these points even when the disclosure is negative.

Sample size	Sample size was not calculated previously, we determined sample size based on the convenience and funding available for the sequencing and genotyping. We had in mind that for this type of work the larger the sample size the better. However, It is important to point out that the BIPMed sample (N = 358), was similar in size to the other datasets used for the comparative analyses performed in the present work, which contained European (N = 404), African (N = 504), and Admixed American (N = 347) populations.
Data exclusions	We remove DNA variants containing > 20% missing data.
Replication	The PCA used to assess the two BIPMed datasets revealed that both WES and SNP array datasets produced similar results.
Randomization	BIPMed participants were identified among people who were accompanying patients in the out-patient clinic of our hospital and were mainly unrelated spouses of patients. We also applied a structured questionnaire regarding serious health issues and excluded individuals that were known to have major health problems.
Blinding	Blinding was not relevant to this study since all calculations were machine-based, no operator influences occurred.

Reporting for specific materials, systems and methods

We require information from authors about some types of materials, experimental systems and methods used in many studies. Here, indicate whether each material, system or method listed is relevant to your study. If you are not sure if a list item applies to your research, read the appropriate section before selecting a response.

Materials & experimental systems

n/a	Involved in the study	
☒	☐	Antibodies
☒	☐	Eukaryotic cell lines
☒	☐	Palaeontology
☒	☐	Animals and other organisms
☐	☒	Human research participants
☒	☐	Clinical data

Methods

n/a	Involved in the study	
☒	☐	ChIP-seq
☒	☐	Flow cytometry
☒	☐	MRI-based neuroimaging

Human research participants

Policy information about studies involving human research participants

Population characteristics	We examined 358 individuals, predominantly from Southeast Brazil (49.44%), at the University of Campinas (UNICAMP, Campinas, Brazil). BIPMed participants were identified among people who were accompanying patients in the out-patient clinic of our hospital and were mainly unrelated spouses of patients.
Recruitment	All participants were volunteers, we offered participation to all accompanying persons of patients at UNICAMP university hospital. Volunteers were only excluded if they have major health problems.
Ethics oversight	The present study was approved by the Research Ethics Committee at UNICAMP, and all participants signed consent forms before participating in the study.

Note that full information on the approval of the study protocol must also be provided in the manuscript.
